# Could prophylactic antivirals reduce dengue incidence in a high-prevalence endemic area?

**DOI:** 10.1371/journal.pntd.0012334

**Published:** 2024-07-29

**Authors:** Yalda Jafari, Oliver J. Brady, Joseph R. Biggs, Le Thuy Lien, Huynh Kim Mai, Hien Anh Thi Nguyen, Marnix Van Loock, Guillermo Herrera-Taracena, Joris Menten, Chihiro Iwasaki, Mizuki Takegata, Noriko Kitamura, Hung Do Thai, Bui Xuan Minh, Kouichi Morita, Dang Duc Anh, Sam Clifford, Kiesha Prem, Julius Hafalla, W. John Edmunds, Lay Myint Yoshida, Martin L. Hibberd, Stéphane Hué

**Affiliations:** 1 Centre for Mathematical Modelling of Infectious Diseases, London School of Hygiene & Tropical Medicine, London, United Kingdom; 2 Department of Infectious Disease Epidemiology, Faculty of Epidemiology and Public Health, London School of Hygiene & Tropical Medicine, London, United Kingdom; 3 Department of Infection Biology, Faculty of Infectious Tropical Diseases, London School of Hygiene & Tropical Medicine, London, United Kingdom; 4 Pasteur Institute in Nha Trang, Nha Trang, Vietnam; 5 National Institute of Hygiene and Epidemiology, Hanoi, Vietnam; 6 Janssen Research & Development, Janssen Pharmaceutica NV, Janssen Pharmaceutical Companies of Johnson & Johnson, Beerse, Belgium; 7 Institute of Tropical Medicine, Nagasaki University, Nagasaki, Japan; 8 Khanh Hoa health Service Department, Nha Trang, Vietnam; University of California Davis School of Veterinary Medicine, UNITED STATES OF AMERICA

## Abstract

Prophylactic drugs against dengue are currently under development. In this study, we explored how such prophylactic approaches might affect dengue cases in four communes of Nha Trang City, Vietnam. A community level dengue transmission survey indicated high levels of previous exposure to dengue (89.7%; 95% CI: 87.2,92.0). We fitted a spatially explicit model to an observed outbreak and simulated likely effectiveness of Case-Area Targeted Interventions (CATI) and One-Time Mass Distribution (OTMD) of drug and vector control strategies. Increasing radius and effectiveness and decreasing delay of CATI was most effective, with drugs being more effective in averting dengue cases than vector control. Using an OTMD approach early in the outbreak required the least number of treatments to avert a case, suggesting that OTMD strategies should be considered as pre-emptive rather than reactive strategies. These findings show that pre-emptive interventions can substantially reduce the burden of dengue outbreaks in endemic settings.

## Author summary

Dengue is a mosquito-transmitted virus that is a leading cause of hospitalisation and death in Asian and Latin American countries. Protection against dengue infection mainly relies on mosquito control, but the current development of drugs against the virus opens new perspectives for prevention. To develop efficient prevention strategies, it is important to understand how prevalent and transmissible the virus is in a given population. In this study, we conducted a large serological survey in Nha Trang City, Vietnam, and developed a mathematical model to simulate the effect of preventive antiviral treatment on dengue outbreaks in that population. We compared the strength of these effects to that of other prevention startegies, such as mosquito control. We found that a large proportion of the population had been previously infected by dengue virus. In this context, our simulations showed that drugs are more effective in preventing dengue cases than mosquito control, and that these strategies are most effective when the radius of intervention increases and the delay of intervention decreases. These findings show that preventive administration of antivirals during outbreaks can substantially reduce the spread of dengue virus in part of the world where it is highly prevalent.

## Introduction

Dengue is a viral disease transmitted through the bite of mosquitos of the *Aedes* genus in tropical and subtropical regions. Several million of dengue infections are reported worldwide yearly, and the virus has become a leading cause of hospitalisation and death in Asian and Latin American countries [1]. Control tools against dengue virus (DENV) are currently very limited. There is no approved pharmaceutical treatment against the virus, and clinical interventions for severe disease are mostly reduced to fluid management [1]. The first vaccine against dengue, Dengvaxia, was licensed in 2015 but is now only recommended for those aged between 9 to 45 years with previous dengue exposure, due to concerns of vaccine-induced increased disease severity in those without previous exposure [2]. Thus, vector control remains the approach of choice for controlling dengue incidence. This includes the elimination of oviposition sites, the culling of larvae and adult mosquitoes, the physical removal of standing waters, and insecticide space or indoor residual spraying [1,3–5]. Most recently, a novel prophylactic approach based on the introduction of mosquitos infected with the bacterium *Wolbachia*, known to reduce vector competence, showed good potential [6–9]. However, given that *Aedes aegypti* mosquitoes are typically active only over distances of 100m or less, these vector control strategies are often effective against infection only in the immediate vicinity of the control method and are resource-consuming [3,5,9–11]. A precise evaluation of vector control methods is difficult [11] but judging from the continual increase of case numbers worldwide, despite their implementation, it seems that vector control has been largely ineffectual at reducing dengue disease burden. Urban, day biting mosquitos have been particularly difficult to control, since bed-nets are inefficient against them. While vector control and *Wolbachia-*reduced vector competence techniques will continue to develop and hopefully improve, new approaches for preventing dengue disease are urgently required.

There is ongoing research on new antiviral compounds that could provide prophylactic and therapeutic benefits against dengue [12,13]. If or when these molecules become available, their efficacy will depend on a precise understanding of the spatiotemporal optimums in applying a drug-based strategy, on its own or in combination with other existing interventions. Such optimisation will be essential to guide public health decisions and policy planning. One option is Mass Drug Administration (MDA), or One-Time Mass Distribution (OTMD) if administrated once at the start of an outbreak, as is employed for malaria transmission interruption in some areas [5,14,15]. Challenges with this approach include the logistic difficulty of treating all individuals residing in an area, the ethical implications of treating non-infected individuals, with the possibility of pharmacological side-effects, as well as the potential emergence of drug resistance [5,14,15]. An alternative approach to MDA or OTMD could be the spatial targeting of interventions in populations surrounding reported cases. This approach has been employed to target incidence hotspots of malaria [16–18] where it has been successful in limiting spread of the outbreaks.

Dengue is endemic in Vietnam, with incidence of cases increasing with proximity to the equator. With an estimated mean burden of 2.6 million symptomatic and 8.0 million asymptomatic dengue cases in 2010, improved control measures could have a significant impact on public health in Vietnam [19]. In Vietnam, dengue follows a seasonal pattern, with higher incidence in the rainy season in the second half of the year [20–22]. This seasonality, together with outbreaks of unpredictable amplitude from one year to the next, a high force of infection (FOI) [20] and low to moderate rates of care seeking [19], calls for reactive, flexible and logistically-manageable control measures. An optimised antiviral-based preventive strategy could become the foundation of such interventions.

In order to better understand the impact of various interventions in mitigating a dengue outbreak in an urban, Vietnamese setting, we have conducted a seroprevalence survey to characterise previous exposure to dengue virus and a three-year clinical observational study. The serosurvey was conducted on a representative sample of the population and we used these detailed epidemiological investigations to parameterise a spatial patch-based stochastic mathematical model [10] to assess the effectiveness of OTMD, anti-viral based and vector control CATI in averting cases during a seasonal outbreak in the area. We identified the most effective approach in curtailing an outbreak following diagnosis of a case and assessed the feasibility of its implementation in real-time outbreak situations using simulations.

## Materials and methods

### Ethics statement

Ethical approval was obtained from the Vietnamese Ministry of Health and London School of Hygiene and Tropical Medicine ethical review boards (IRB-VN01057-27/2015). Written, informed consent was obtained from participants over 16 years; for individuals under 16 years, written consent was obtained from parents/guardians. The objectives of the survey were explained clearly to participants. Participation was voluntary and individuals had the opportunity to opt-out at any point during the interview process.

### Serosurvey

A stratified serosurvey was conducted in the Vinh Hai and Vinh Phuoc communes of Nha Trang City, Vietnam, in June 2017. In each commune, a random sample of 50 individuals listed in a census established in 2015 was drawn from each of the following age categories: 6 months to 5 years, 6 to 15 years, 16 to 25 years, 26 to 35 years, and 36 to 55 years.

Study teams visited each selected individual at their home. If the individual was not at home, a second attempt was made to reach the individual within one week. If the individual was not reachable on the second attempt, that person was considered lost to follow-up. Those who agreed to participate in the study were visited at their homes for further interview and blood sample collection. Longitude and latitude of the participants’ household address were also recorded.

At the commune health centre, survey teams interviewed each individual using a standardised questionnaire collecting information on demographic and social characteristics including age, sex, head of household, education level of the recruited individual and of the head of household, occupation, vaccination history, and illness record in the past two weeks. A venous blood sample of 5 mL for adults and 2 mL for children under 5 years was drawn from each participant and plasma was stored at -80°C prior to assay. The blood samples were processed and tested at the Pasteur Institute in Nha Trang.

Study participant serum was tested for anti-DENV IgG (immunoglobulin G) antibodies using Panbio capture ELISA tests [23,24] according to manufacturer’s specifications. The commercial cut-offs for these tests are designed for the detection of active dengue infections. Since this study was concerned with determining exposure to dengue at any point in an individual’s lifetime, we utilized a previously generated IgG seroprevalence threshold of 2.2 panbio units indicative of previous dengue exposure, generated using a mixture modelling approach and identical ELISA kits [25]. Individuals with IgG panbio units above this threshold were classified as seropositive and having had previous exposure to dengue.

We assumed that the probability of selecting each individual within each age group was equal to the number of individuals selected divided by the size of that age group in the study area, corresponding to a statistical weight. This weight represented the number of individuals each sampled individual represented. The population prevalence of DENV-specific IgG antibodies was calculated using the *survey* package in R [26]. A bootstrapping approach was used to account for variability due to sample selection, using 10,000 replications.

### Force of infection estimation

To estimate long-term average transmission intensity, dengue force of infection was estimated using two of the studied communes, namely Vinh Hai and Vinh Phuoc. A catalytic model was fit to the seroprevalence data from each commune using the *rjags* R package [27], run with two chains of 100,000 iterations, and thinned at every 10^th^ iteration with a burn-in of 20,000. Convergence was confirmed visually and with a Heidelberg test [27].

The force of infection was calculated using the following relationship. The serostatus of each individual, *y*_*i*_, at age, *a*_*i*_, was deemed to follow a Bernoulli distribution,

$$y_{i}=Bern(p_{i})$$

pi=1−e^{−λai}

where *p*_*a*_ is the probability of seropositivity at a given age and *λ* the force of infection. The parameter *λ* had a weakly informative prior, *U*(0, 1), while age (*a*_*i*_) was included as a continuous variable.

The risk of previous exposure by risk factors, after adjusting for risk by age using the force of infection relationship, was investigated using a generalised linear model, adjusting for survey sampling using the *survey* package.

Using the calculated force of infection, the long-term average incidence of dengue infection was estimated using the formula established by Salje *et al*. [28]. We assumed that all four dengue serotypes circulated and contributed equally to the force of infection in the survey area. The estimated number of primary, secondary, tertiary, and quaternary infections was then calculated as follows:

w(a)=4e^{−3λsa}{1−e^{−λsa}}

w2(a)=6e^{−2λsa}{1−e^{λsa}}2

w3(a)=4e^{−λsa}{1−e^{−λsa}}3

Numberofprimaryinfections=N(a)4λse^{−4λsa}

$$Number\;of\;secondary\;infections=N(a)3\lambda_{s}w(a)$$

$$Number\;of\;tertiary\;infections=N(a)2\lambda_{s}w2(a)$$

$$Number\;of\;quaternary\;infections=N(a)\lambda_{s}w3(a)$$

where *w*(*a*), *w*2(*a*), and *w*3(*a*) are the proportion of the population that had already been infected with one serotype, two serotypes and three serotypes in previous years, respectively, and *N*(*a*) the number of individuals in the age group. An estimate of the number of expected cases per 100,000 was obtained using the expected proportion and number of individuals in each age group using census data collected in 2015. The incidence of reported cases was calculated using the enrolled dengue case data and the aforementioned census. The notification rate was calculated as the proportion of expected cases that reported to either a clinic or hospital.

A geospatial analysis of the seroprevalence data was conducted to determine the spatial distribution of immunity to dengue in the study area. Outcome was presence of dengue specific IgG antibodies, as present (0) or absent (1). Data on population count [29], elevation (in meters) [30], Visible Infrared Imaging Radiometer Suite night-time lights [31], distance to major OpenStreetMap waterways (in kilometers) [32], distance to International Union for Conservation of Nature strict nature reserve and wilderness area edges (in kilometers) [33], and topographic slope (in degrees) [34] were extracted from publicly available sources. The resolution of all covariates was at a scale of 100m x 100m. A univariate analysis of each spatial explanatory covariates and outcome was conducted. Variables found to be significantly correlated with the outcome were investigated, both as linear functions and as smoothing splines, using a general additive model (GAM) framework [27]. Geospatial models were also compared with and without geospatial coordinates with a gaussian smoothing function. The model with the lowest AIC was selected and then used to map the spatial distribution of previous exposure to dengue in all communes of interest [27].

### Clinical observation study

Individuals of all ages who resided in the communes of Vinh Hai, Vinh Phuoc, Vinh Tho, and Vinh Hoa in Nha Trang City, Vietnam, and who visited either a partner local Polyclinic or the city’s Tropical Medicine Hospital, were eligible for the study. Local polyclinics are government-lead clinics each covering 5 to 6 city communes. The residents are required to use the designated polyclinic in order to receive optimal government health insurance support. Under the infectious disease control law currently in practice in Vietnam, all dengue positive cases with illness should be referred to the Tropical Medicine Hospital. In the polyclinic, those who presented with fever were enrolled in the study and subsequently tested for dengue with an NS1 rapid test (DENGUE NS1 AG STRIP, #70700, BioRad [35]). In the hospital, only those who were diagnosed with dengue fever on the basis of an NS1 rapid test were enrolled in the study. Enrollment was conducted between October 2016 and May 2019 at the polyclinic and between December 2016 and April 2019 at the hospital. A questionnaire was administered to each individual patient enrolled in the study.

Since DENV is endemic in Nha Trang City, Vietnam, new dengue cases are diagnosed year-round. This makes the identification of the start and end of a seasonal outbreak, where the number of cases sharply increases, difficult to define. A Markov switching model was therefore used on the dengue enrolment data to retrospectively estimate the most likely outbreak period in 2018–19, using the MSwM package in R [36].

### Mathematical model specification

We adapted a patch-based meta-population dengue model developed by Brady *et al*. [10] to investigate the effectiveness of One-Time Mass Distribution approaches (OTMD) or CATI strategies based on either vector control or antiviral-based prophylaxis in Nha Trang. In this study, CATIs were defined as a case-area targeted interventions of varying size and response time. More details on the model structure, assumed underlying transmission dynamics, parameters and priors, initial modelling conditions, and fitting methods can be found in Brady *et al*. [10]. In brief, the model assumes a single-serotype outbreak and uses an SIR-SEI framework: while the human population is divided into Susceptible, Infectious (with time-varying infectiousness) and Recovered compartments (SIR), the mosquito population is classified as either Susceptible, Exposed or Infectious (SEI). Humans are assumed to apportion their time in a number of 100m x 100m patches with degree and directionality of movement fitted to the patterns observed in the dengue observational study data. In the model, spatial heterogeneity between patches is a product of varying immunity and the degree of correlation between immunity and mosquito-human contact rates.

Human population estimates per 100m x 100m pixel were extracted from WorldPop and rounded to the nearest integer. The number of susceptible individuals in each patch was calculated based on a spatial distribution of the proportion of the population previously exposed to dengue, as determined from the serosurvey, mapped to 100m x 100m patches. The following parameters were considered fixed: intrinsic and extrinsic incubation periods of the virus, cumulative human infectiousness over the course of an infection, maximum duration of symptomatic illness following completion of intrinsic incubation period and the average effective reproduction number at the beginning of the outbreak. The distance decay parameters controlling human movement, daily mortality rate of a mosquito, vector-to-human and human-to-vector transmission rate, and type of human model (radiation, gravity, or exponential) were inferred from fitting data to the model. These parameters are summarised in Table 1. Mean human-vector contact rate was adjusted for seasonality inferred from monthly incidence rate ratios, starting at the day of the year corresponding to the inferred start of the seasonal outbreak [22].

**Table 1:** Fixed and fitted parameters used in transmission model.

Parameter	Definition	Value	Reference
**Fixed parameters and constraints**			
**1/*ε***_**h**_	Intrinsic incubation period of the virus	LogNormal(*μ* = 5.9, *σ* = 1.05)	[10,38]
**1/*ε***_***m***_	Extrinsic incubation period of the virus	LogNormal(*μ* = 7, *σ* = 1.23)	[10,38]
***θ***	Cumulative human infectiousness over the course of an infection	$\theta =\int \frac{negBin(29.37,0.88)}{max(negBin(29.37,0.88))}$	[10,39]
**1/*γ***	Maximum duration of symptomatic illness following completion of IIP	8 days	[1,10]
***R***_***t*,0**_	Average effective reproduction number at the beginning of the outbreak	1<*R*_*t*_,_0_<10	[10,40]
**Fitted parameters with priors**			
***k***	Distance decay parameter controlling human movement	U(0,1)	[10]
***δ***	Daily probability of a DENV infected individual being detected and reported as a case	LogNormal(*μ* = −2.6 *σ* = 0.02) /8	[10]
***μ***_***n***_	Daily mortality rate of a mosquito	Beta(*σ* = 11.93, *β* = 107.4)	[10,41]
***β***	Vector-to-human and human-to-vector transmission rate, broken down into mean and spatial correlation components	μ=U(0.1,2) p=U(−1,1)	[10]
***τ***	Type of human movement model	“exponential”, “gravity”, or “radiation”	[10]

The model was fitted to the clinical observation study data using an Approximate Bayesian Computation based on Sequential Monte Carlo (ABC SMC) method [10,37] for three rounds. At each round, 4,000 different parameter samples were taken and evaluated against the mean of 10 model simulations. The priors for the first round were selected from **Table 1**, while for rounds two and three, top 10% parameter sample combinations were used. The model was fitted to data collected during the 2018–2019 outbreak. We define the ourbreak start and stop times by retrospectively analysing case count data using a Markov switching model that identifies the time periods during which variation in case counts significantly differ. The CATI model was initiated using the observed data two weeks prior to the start of the simulated outbreak. The fit of the model under the three movement models (exponential, gravity, and radiation) was assessed using a deviation criterion that scored model performance based on relative distribution of predicted and observed cases over space and time (see model description section in S1 Apprendix). The fitted CATI model was then simulated from the beginning of the outbreak start date. Vector control was assumed to act on adult female mosquitoes (e.g. insecticides) with effectiveness corresponding to the percentage reduction in the adult female mosquito population. Prophylactic drugs were assumed to temporarily (up to 30 days) prevent or cure dengue virus infection in humans and were assumed to have equal efficacy against all serotypes and genotypes of dengue virus. Our goal in this analysis was to explore the relative merits of CATI strategies with vector control vs CATI strategies with prophylactic drugs. To achieve this we standardised or co-varied a number of parameters of each intervention including timing of implementation (start of outbreak, peak or mid outbreak, and late in outbreak), effectiveness, delays in implementation (days between case detection and CATI implementation), and radii of intervention on proportion of dengue cases averted. Any patch with a centroid-to-centroid distance less than or equal to the assigned radii of the intervention received CATI treatment. When the assigned radius was less than 100m, it was assumed that CATI was only implemented in the same patch as the index case. The simulations were ran for 365 days with total cases compared to identify strategies that prevented cases as opposed to those that merely delayed cases until later in the year. We also compare the effectiveness and resource requirements (number of individuals treated with the intervention) of CATI vs an alternative mass distribution approach where every individual in the study area is treated once at the beginning middle or end of the epidemic. This One-Time Distribution approach was tested using both drug and vector control.

## Results

### Previous dengue exposure estimation

**Fig 1:**
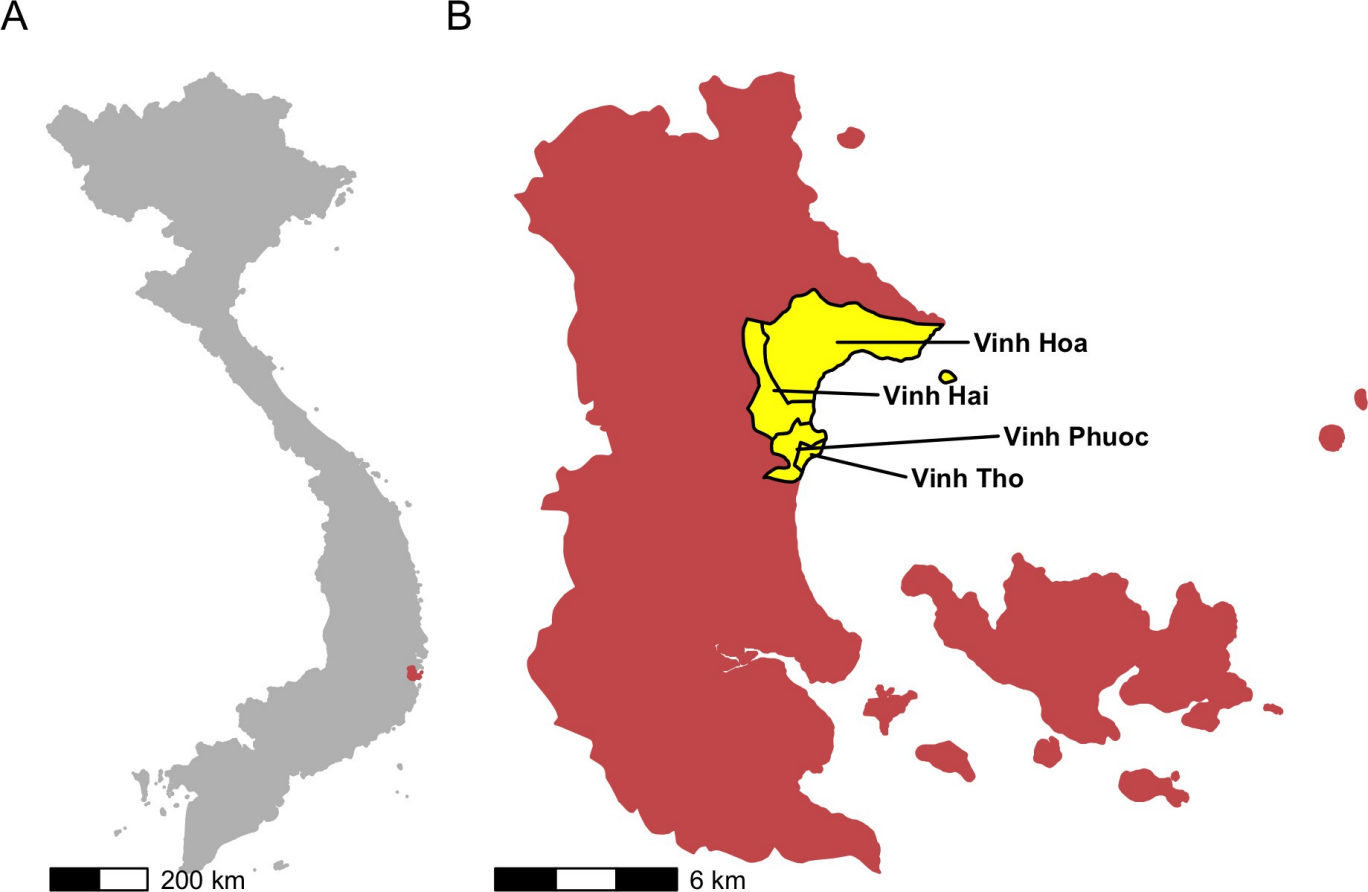
Map of Vietnam in grey with Nha Trang City in orange (A); Map of Nha Trang City with the four communes of interest, Vinh Hai, Vinh Phuoc, Vinh Tho, and Vinh Hoa, highlighted in yellow (B). Source of Administrative boundaries: The Global Administrative Unit Layers (GAUL) dataset, implemented by FAO within the CountrySTAT and Agricultural Market Information System (AMIS) projects.

A seroprevalence survey was undertaken in the Vinh Hai and Vinh Phuoc communes of Nha Trang, Vietnam (**Fig 1**) in June 2017. A total of 510 individuals were surveyed and 508 of these individuals were included in the final analysis, with two individuals excluded because the recorded residence location was outside the geographical boundaries of the Vinh Hai and Vinh Phuoc communes.

**Fig 2:**
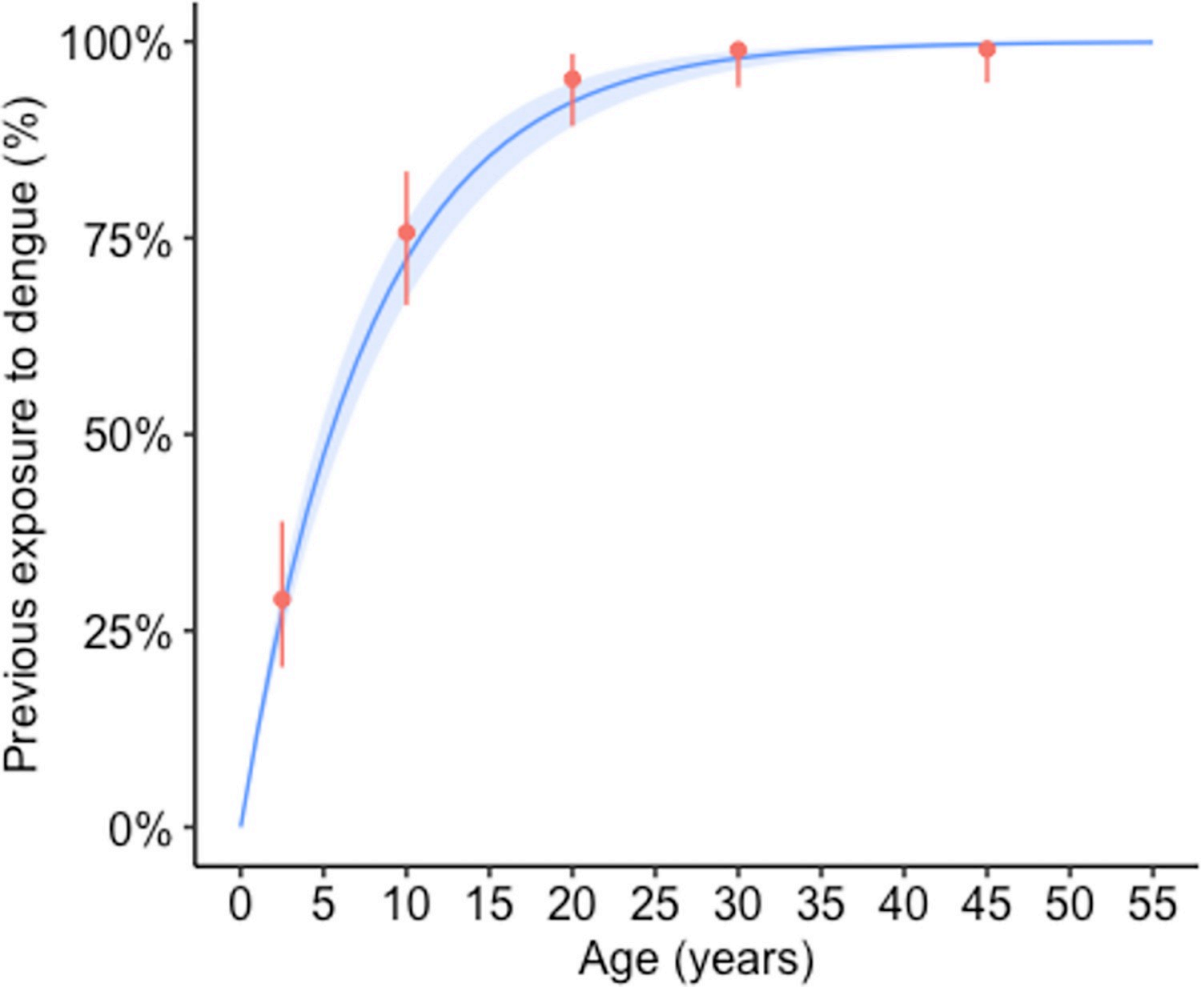
Predicted age-seroprevalence of dengue virus using the catalytic model (blue), and the observed age-seroprevalence with associated confidence intervals (red).

The population-wide weighted prevalence of previous exposure to dengue, as measured by presence of anti-DENV IgG antibodies, was estimated to be 89.4% (95% CI: 87.0, 92.0) in Vinh Hai, 89.7% (95% CI: 87.2, 92.0) in Vinh Phuoc, and 89.6% (95% CI: 87.6, 91.0) in both communes combined. As expected, the prevalence of previous exposure to dengue was highest in the oldest age groups. After a sharp exponential increase in individuals aged 0 to 20 years, prevalence plateaued around 99.0% (95% CI: 94.9, 100) amongst individuals between 36 to 55 years (**Fig 2**).

The mean dengue force of infection was estimated as 0.14 (95% CrI: 0.10, 0.15) in Vinh Hai and 0.14 (95% CrI: 0.12, 0.18) in Vinh Phuoc. The FOI in the two communes was not statistically different as the credible intervals overlapped. A spatial model was fit to data collected from two of the studied communes in order to estimate spatial variation of dengue seroprevalence. Using the geospatial covariates described in methods and an automatic variable selection option in gam, models using splines and no splines, as well as without any covariates were assessed. The model with no covariates had the lowest AIC and was therefore selected. No spatial pattern was found in the serostatus data, and a spatially constant longterm (year-to-year) previous exposure to DENV was assumed over the four communes of interest in Nha Trang City.

Based on the above findings, the data from the two communes were combined to estimate an overall annual FOI of 0.13 (95% CrI: 0.11, 0.14) over the two sampled communes (**Fig 2**). We found the estimated incidence of expected DENV infections to be 5,250 per 100,000. Based on these predictions, we calculated the percentage of estimated infections that reported to our recruitment sites, the DENV infection detection rate, to be 7.8% (95% CI: 7.5, 8.2). Residual risk of risk factors was investigated after removing the risk of seropositivity associated with age. For all variables considered, the confidence interval of the residual crossed zero, suggesting that none of the tested variables (sex, occupation of head of household, education of head of household, and position of head of household within the household), were significant risk factors in seropositivity. Results of this analysis can be found in **S1 Table**.

### Observed outbreak

Between October 2016 and April 2019, a total of 2,287 residents of the 4 study communes were recruited for a clinical observation study. Of these, 1,791 were enrolled at the Polyclinic No.2 and 496 at the Tropical Medicine Hospital, Khanh Hoa Province. A total of 839 NS1 positive dengue patients were identified, ten of which (1.2%) were excluded as their household was outside the study region.

**Fig 3:**
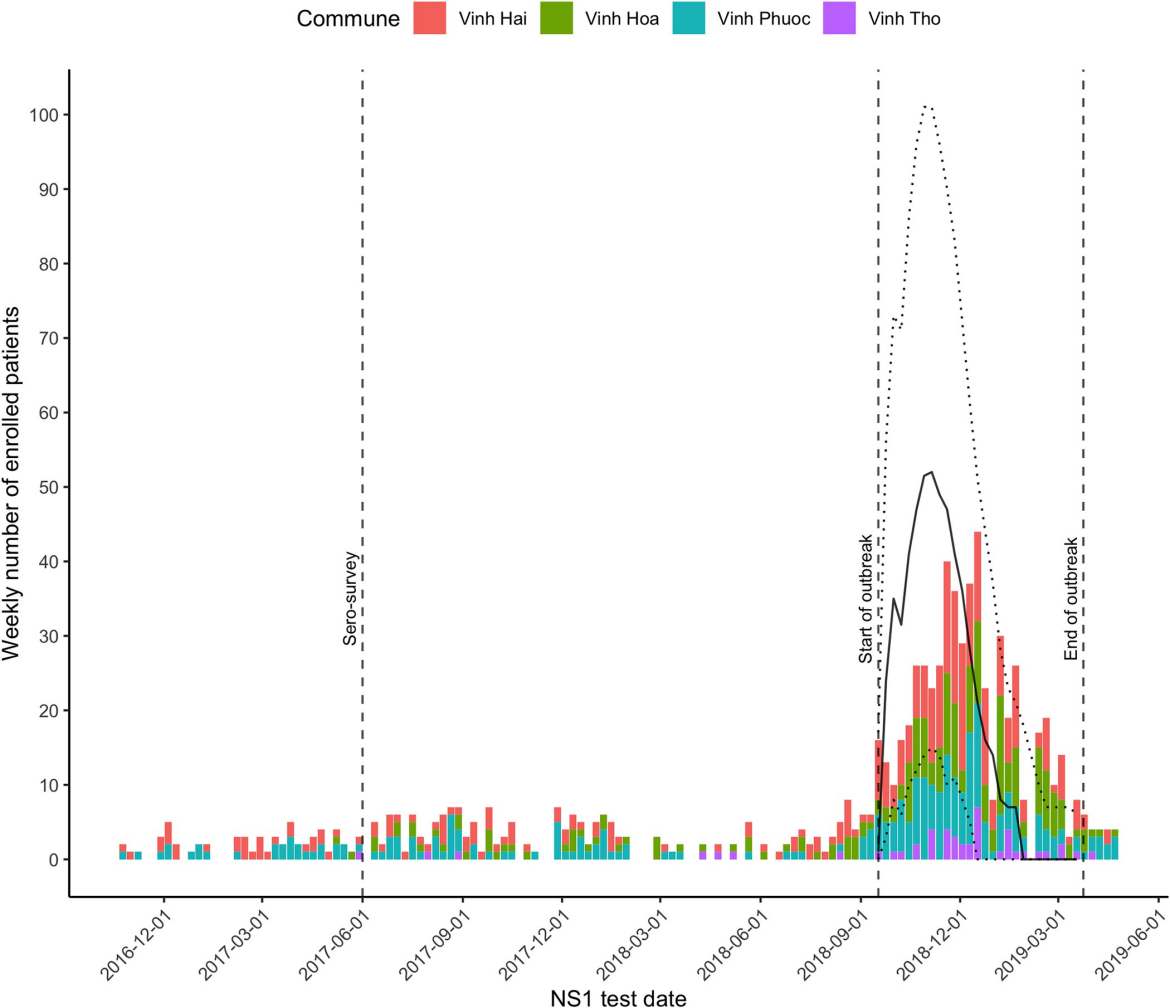
Number of dengue positive patients enrolled per month and per commune between October 2016 and May 2019. The vertical dashed lines represent the date the serosurvey (June 2017), and the boundaries of the observed outbreak (September 17, 2018 to March 24, 2019). The plain and dotted black curves represent the model-predicted mean number of cases and confidence intervals, respectively.

Of the 829 cases, 57.4% were male. The median age was 18 years (IQR: 9.0 years– 26.2 years). Over the study period, the annual incidence rate in the cohort was highest in the Vinh Hai commune, with 171.4 cases per 100,000, followed by Vinh Hoa (136.7 cases per 100,000), Vinh Phuoc (120.8 cases per 100,000) and Vinh Tho (72.0 cases per 100,000). The number of dengue positive patients started increasing in July 2018 with the highest number in the third week of December 2018 (**Fig 3**). Using a Markov switching model to distinguish the outbreak period from baseline endemic levels of infection, the outbreak in the four communes was estimated to occur between September 17, 2018 and March 24, 2019. The epidemic curve of enrolled cases is presented in **Fig 3**.

### Human movement model testing

To estimate the effectiveness of potential intervention strategies, a spatially explicit dengue transmission model was informed by long-term average FOI estimates and dengue detection rates, with this data then fitted to the dengue patient data. To estimate human movement behaviours, we compared the fit of three different human movement models to determine which would provide the best fit to the spatial patterns in the patient data. These models were fit to the patient data from September 2018 to March 2019 (**Fig 3**), under three different movement assumptions: exponential, gravity, and radiation. Three rounds of Sequential Monte Carlo Approximate Bayesian Computation were run. Each consecutive ABC SMC round provided an improved fit of the model to the data, ranging from 33.8% using a radiation movement to 41.9% using an exponential model. The final deviation metric between model predictions and the data was similar amongst the different movement models, ranging from 0.4 in the exponential and gravity models to 0.48 in the radiation model, (**S2 Table**).

The posterior distribution of parameters by movement type suggested similar transmission dynamics amongst the three movement models (**S3 Table and S1 Fig**). All three movement models suggested that infected individuals were likely to spend approximately less than a quarter of their time at risk concentrated around their home (during the day time, when they are at risk of being bitten). Mean human-vector contact rate was also similar across the three movement models with the cases showing some correlation with historical DENV exposure levels under the Gravity model (95% CrI: 0.48 (-1.0, 0.93)) but very small correlation in Exponential (0.080 (95% CrI: -0.94, 0.99)) and Radiation (0.021 (95% CrI: -0.98, 0.97)) models. Credible intervals of all the parameters overlapped when comparing results between movement types, suggesting that all three movement models can be used to explain population mobility in our study.

Posteriors for each parameter was used to estimate mean number of DENV cases per 100m by 100m patch (**S2 Fig**). Within each patch, the week during which the peak of the outbreak was reached was compared across movement models and with the observational data collected during the outbreak (**S3 Fig**). The peak was reached slightly earlier in the radiation model compared to the other models and data.

**Fig 4:**
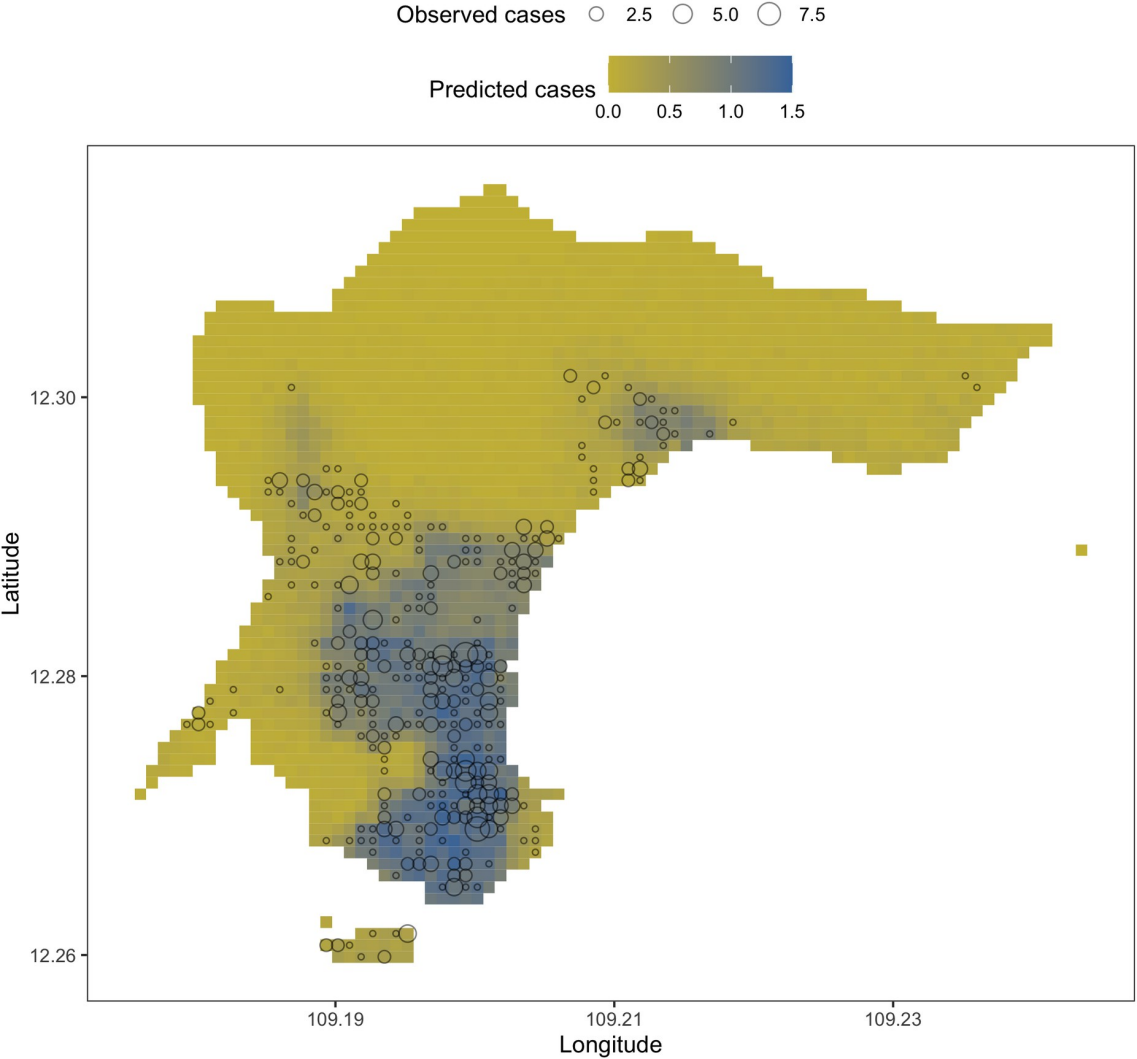
Spatial distribution of observed and predicted dengue cases by patch (100mx100m) across the four communes during the observed outbreak, using an ensemble of posterior distributions.

As there was no significant difference between the movement models in final deviation and posterior parameters, all three sets of posterior parameters under the three movement model assumptions were combined in an ensemble in the transmission model (**Fig 4**). Each set was weighted according to its deviation.

### CATI effectiveness assessment

**Fig 5:**
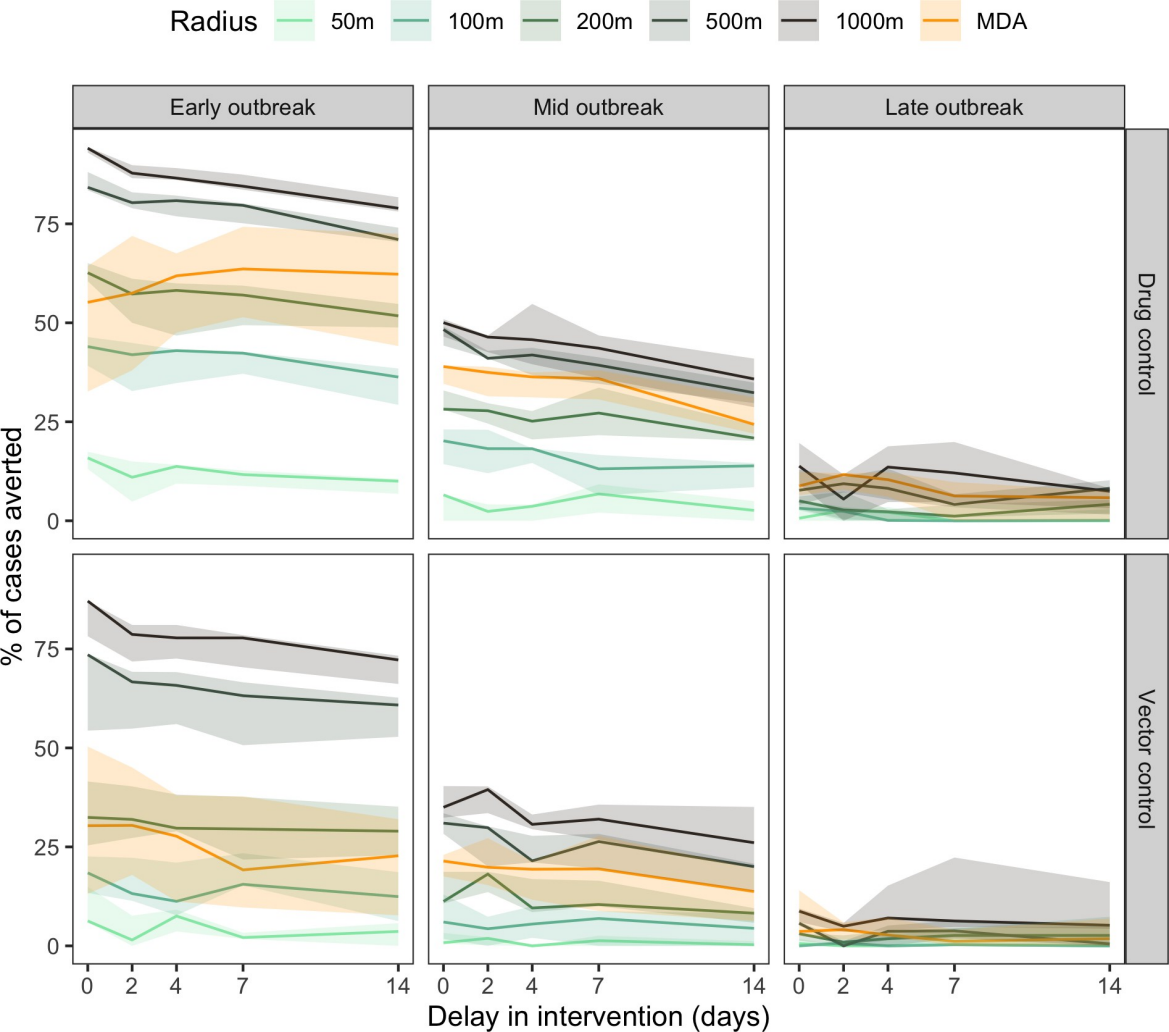
Percentage of cases averted, by delay in intervention (days), assuming constant effective coverage (0.81) and intervention length (180 days); by type of intervention (Drug control, Vector control), time of application in outbreak (Early (outbreak day = 1), Mid (outbreak day = 52), Late (outbreak day = 92)), and radius of CATI and One-time mass distribution (OTMD).

**Fig 6:**
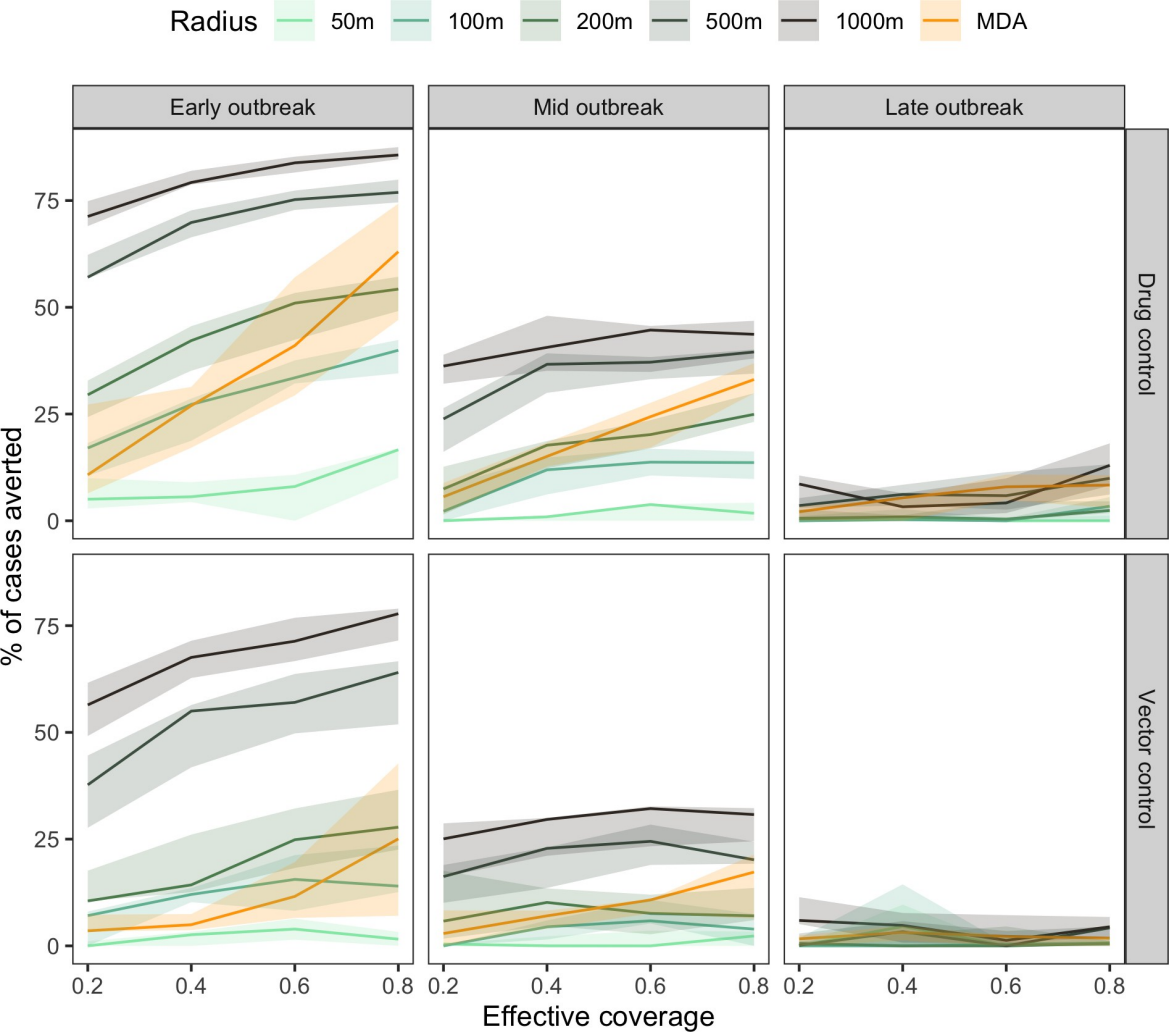
Percentage of cases averted by change in effective coverage, assuming constant delay (7 days) and intervention length (180 days) by type of intervention (Drug control, Vector control), time of application in outbreak (Early (day = 1), Mid (day = 52), Late (day = 92)), and radius of CATI and One-time mass distribution (OTMD).

Model simulations were run to investigate the capacity of drug-based and vector control strategies to reduce dengue cases under various realistic assumptions. The parameters tested included (i) effective intervention coverage, (ii) intervention delays, (iii) size of the intervention area (including OTMD), and (iv) stage of the outbreak (early, mid, or late) at the time the intervention is initiated (**Figs 5** and **6**). Early outbreak was defined as the first day of the outbreak, mid outbreak was defined as the day when the peak of the outbreak was reached (approximately day 52), and late outbreak was defined as half point between peak and end of outbreak (approximately day 92) (**Figs 3** and **S4**).

The impact of intervention delays and intervention area size on the percentage of cases averted was assessed for an effective coverage fixed to 81% (**Fig 5**). An effective coverage of 81% was used to represent 90% of individuals receiving an intervention, drug or vector control, that is 90% effective. Increasing the delay in intervention deployment resulted in decreases in the percentage of cases averted (**Fig 5**). The highest percentage of cases averted was achieved using a CATI strategy at 1000m x 1000m on the day of diagnosis of the first case (i.e. 0 days of delay), early in the outbreak. Applying a drug intervention at the mid outbreak at 1000m resulted in percentage of averted cases similar to a 200m CATI intervention early in the outbreak. The highest percentage of cases averted when applying intervention late in the outbreak was only as high as applying a 50m CATI strategy early in the outbreak. Similar trends were observed for a vector-control intervention, though drug control resulted in higher percentage of cases averted. OTMD intervention, i.e. treating all individuals in one day, was more effective than drug-based CATI at 50m or 100m for 180 days, but performed consistently less well than intervention in patch sizes greater than 500m.

Percentage of cases averted as a result of changing effective coverage and a constant delay of seven days was assessed (**Fig 6**) to reflect realistic scenarios, noting that small delays around this period led to only small differences. Increasing effective coverage resulted in an increase in cases averted in all simulations, with higher percentages of cases averted the earlier the intervention was deployed in the outbreak, together with higher radius of drug administration.

OTMD compared to CATI treatments was variable depending on the effective coverage. An early OTMD intervention of effective coverage of 20% was only better than a 50m CATI intervention while an OTMD of effective of 80% was better than a 200m CATI intervention. In all situations, drug control averted more cases than vector control, given the same conditions.

**Fig 7:**
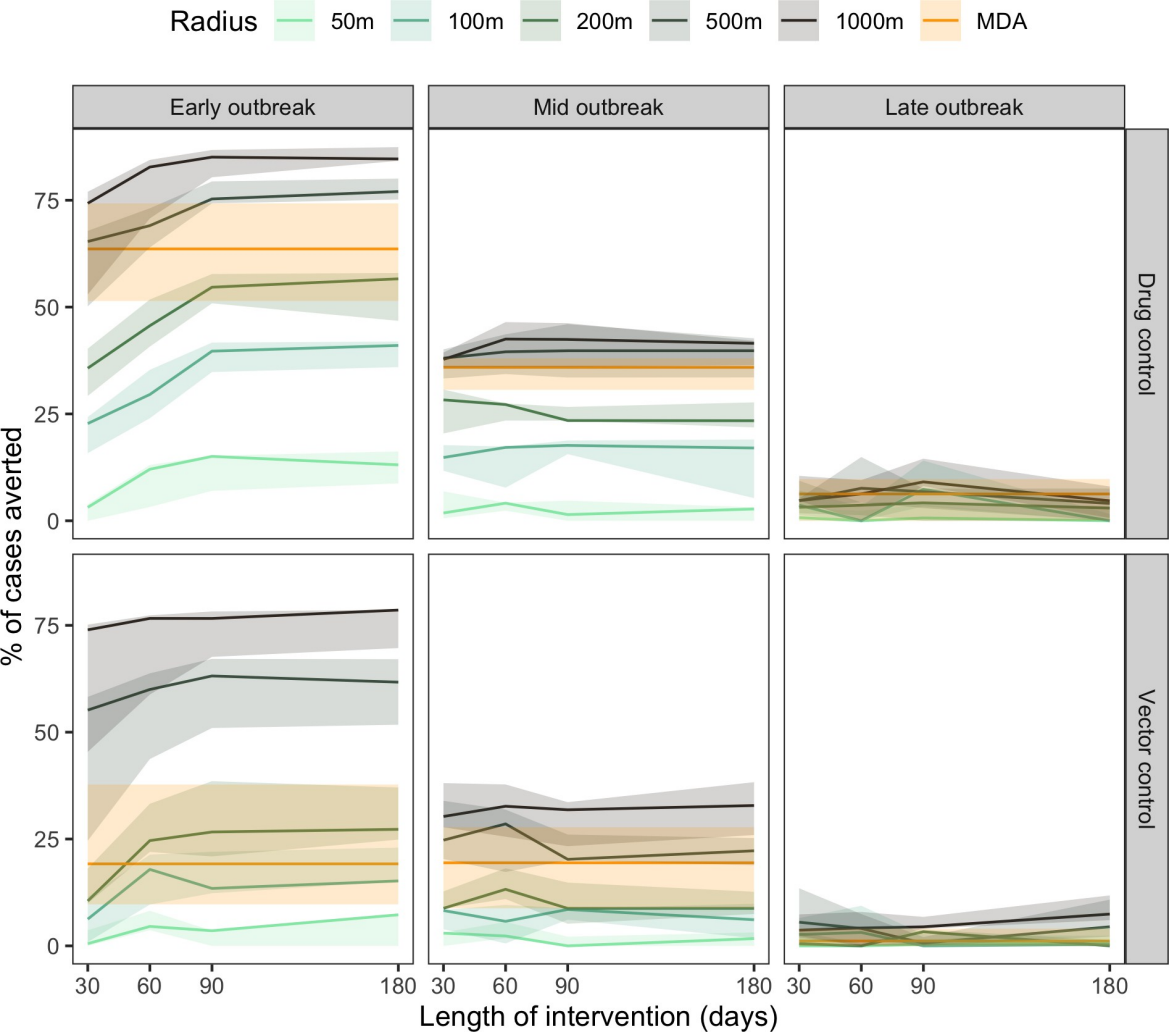
Percentage of cases averted, by length of application of intervention (days), assuming constant effective coverage (0.81) and delay (7 days) by type of intervention (Drug control, Vector control), time of application in outbreak (Early (day = 1), Mid (day = 52), Late (day = 92)), and radius of CATI and One-time mass distribution (OTMD).

The effect of the length of the drug intervention period, starting at different phases in the outbreak, on the number of cases averted was investigated (**Fig 7**). When the intervention was initiated at the start of the outbreak, the percentage of cases averted increased with the length of drug administration up to 90 days of intervention, after which no significant improvement on the percentage of averted cases was observed. However, the duration of the drug intervention had no significant impact on the percentage of cases averted when initiated during mid or late outbreak. In a 1000m CATI strategy, applied early in the outbreak, the percentage of cases averted at 60 days was 82.8% (CI: 70.8, 84.4). This percentage increased by only a small amount to 84.7% (CI: 84.2, 87.4) at day 180. A similar pattern was observed with vector control-based interventions.

**Fig 8:**
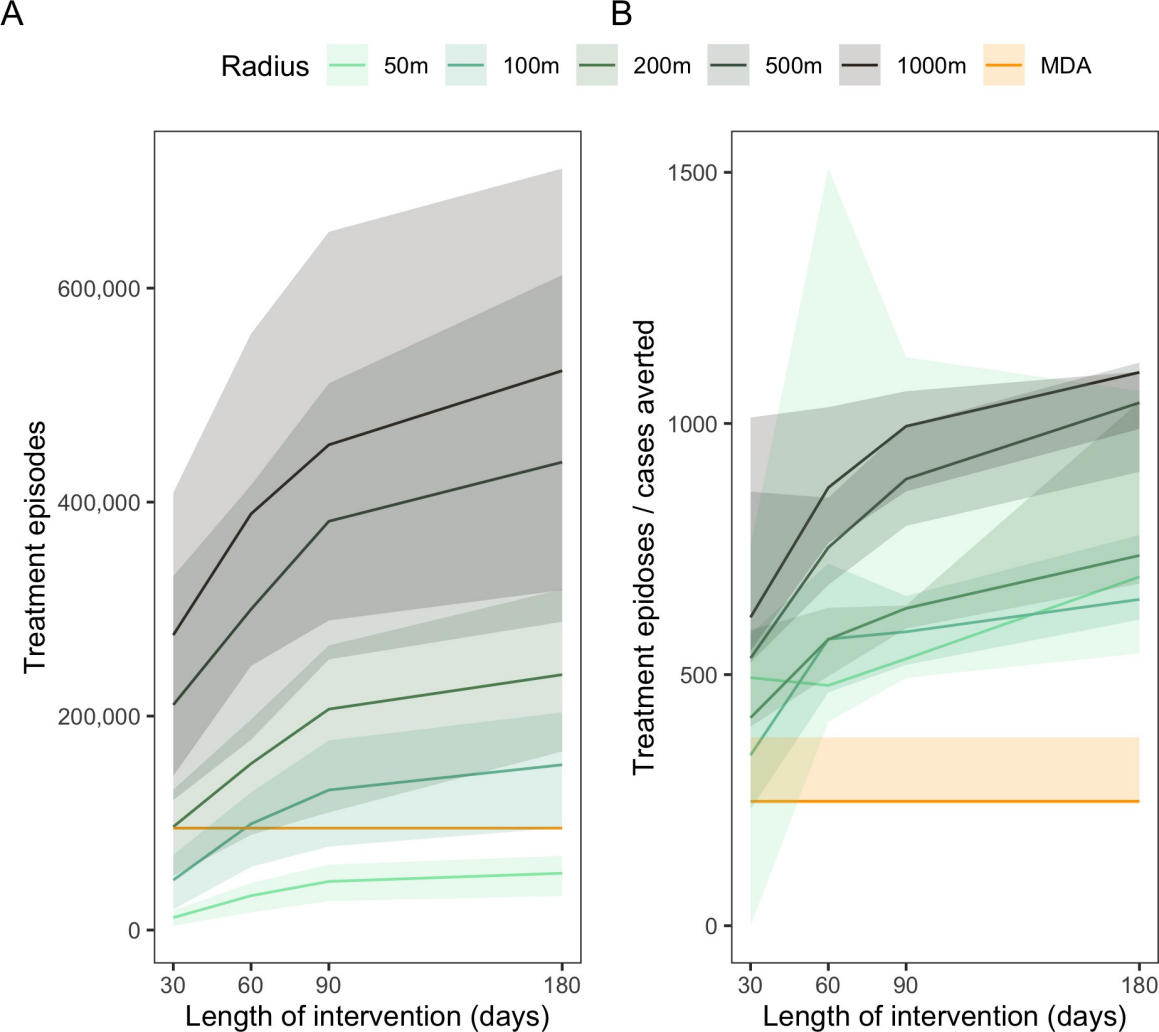
Number of anti-viral episodes with drug (A) and ratio of episodes to averted cases(B), assuming constant effective coverage (0.81) and delay (7 days), applied early (day = 1) in an outbreak.

We next looked at the number of anti-viral episodes each strategy would require to significantly impact the number of subsequent cases. As expected, the number of required doses increases sharply as the treated area and, to a lesser degree, the length of duration of the intervention increase (**Fig 8A**). For instance, a 50m CATI strategy will require 53,137 doses (CI: 31,889, 69,385), if carried out for 180 days. Meanwhile, a 1000m CATI strategy implemented for a period of 90 days requires the largest number of anti-viral episodes: 453,540 doses (CI: 289,370, 652,651). By contrast, an OTMD approach would require 95,266 doses in one intervention, making this strategy the second least drug-consuming of all tested strategies, after 50m x 50m patch interventions.

To explore the cost-benefit relationship between the different strategies, we examined the number of drug episodes required to avert a dengue case (**Fig 8B**). The OTMD approach, with only one dosing episode per person, had the lowest ratio, at 247.8 (CI: 242.7, 375.1); while the 1000m CATI strategy with an intervention length of 180 days had the highest ratio, 1,101.9 (CI: 989.2, 1,103.3), indicating higher number of individuals are needed to be dosed prophylactically to avert a case of DENV.

## Discussion

Dengue, a mosquito-borne viral disease, has swiftly spread worldwide in recent years. Novel antiviral compounds are being developed against dengue. By conducting a seroprevalence study coupled to stochastic mathematical modelling, we show that an effective antiviral compound could be used prophylactically to help reduce the number of reported dengue cases in an endemic area, during an outbreak situation.

The seroprevalence survey we conducted in two communes of Nha Trang City in 2017 determined that 89.9% (95% CI: 87.9, 92.0) of the tested population aged 6 months to 55 years were previously exposed to dengue. A cross-sectional study conducted in children aged 1 to 10 years in Nha Trang City, in 2015, found that about one third of children were previously exposed to dengue [20]. These two findings are in line with each other, supporting the conclusion that there is a high level of background transmission intensity of dengue infection in the area, with about 90% of the population having been exposed to dengue at least once by the age of 25. We did not find support for a spatial pattern in background immunity.

We investigated the predicted effect of drug- and vector control-based CATI strategies, as well as mass drug administration, on controlling new infections. Our study provides evidence that a CATI prophylactic approach would be an effective course of action to reduce the number of dengue reported cases during an outbreak, by up to 87%. CATI strategies were most effective when applied within the first two days of identifying an index case. Increasing the size of the intervention helped counteract the effect of both intervention delays and limited effective coverage. Comparing drug or vector control-based CATI strategies, the antiviral CATIs were more effective in averting cases.

We found that increasing the interventions up to a radius of 1000m patch counterbalanced delays in response and decrease in effective CATI coverage. Prophylactically treating individuals within an area of 1000m x 1000m of each reported case for 180 days, which resulted in the highest percentage of cases averted, 84.7% (CI: 84.2, 87.4), would mean using around 522,814 (95% CI: 317,739, 711,811) anti-viral episodes in a population of 95,266 people. This may be logistically difficult to implement, therefore we explored alternative strategies. For example, drug intervention over an area of 2000m x 2000m for 90 days, using about 206,483 (95% CI: 109,946, 265,993) dosing episodes with 81% effective coverage conducted 7 days after index case identification would avert 54.6% (95% CI: 50.9, 57.7) of cases, which would be more effective than an equivalent mosquito control intervention at 26.6.% (95% CI: 20.9, 38.5) of cases averted.

Another strategy we considered was OTMD of drugs. While the percentage of cases averted was not the highest with this strategy, the number of individuals required to treat in order to prevent a case was the lowest. This finding is in line with our observation that there is no spatial pattern in previous exposure to DENV in the area, as well as the low estimate in the spatial correlation component of human-vector contact rate, indicating low correlation between outbreak cases and previous DENV cases. Considering the evidence, the recommended intervention in this setting would be an OTMD applied early in the outbreak, provided that this can be accurately and reliably detected. CATI approaches can still be considered until an outbreak is declared.

The low effectiveness of vector control in our study is consistent with previous reports where effectiveness of chemical control of vectors were found to be low or variable at best [4,42–44]. Our findings suggest that a prophylactic drug approach would outperform an intervention based on vector-control only. A quicker, smaller scale intervention may have similar effects as a delayed, lager scale intervention. Improved diagnostics, surveillance, and preparedness can reduce delay in response, thus reducing the need for larger scale interventions.

Dengue in central Vietnam was found to follow a seasonal trend, with higher incidence of cases observed in the rainy season, from May to December [20,22]. We accounted for seasonality using monthly incidence rate ratios of dengue infections in Khanh Hoa province between 1994 and 2013 [22]. We assumed incidence rate ratios were the same every year despite evidence of high interannual variability in dengue cases in Nha Trang City [20].

While we followed rigorous procedures to fit our model to data and to capture uncertainty at various steps of our analysis, there were several limitations to our study. The serosurvey was completed in two communes (Vinh Hai and Vinh Phuoc) while the patients of the prospective study were enrolled from four communes. This required us to extrapolate our findings from the serosurvey to the communes, Vinh Tho and Vin Hoa, which may introduce selection bias.

The observed dengue incidence data was from patients that visited a local Polyclinic or Tropical Medicine Hospital. We will have missed some residents who visited other private clinics and hospitals both within and outside the geographical area of interest. This would result in an underestimation of the number of reporting cases in the area. Dengue Ag rapid testing is covered by health insurance and under infectious diseases control law in Vietnam, all dengue positive cases should be referred to Tropical Medicine Hospital. Therefore, the proportion of missed cases should be small. A limitation of the specific NS1 rapid test that was used in our study is that using this test alone can miss dengue infections, particularly secondary dengue infections.

Population data obtained from our team’s previous census study and from WorldPop were not identical. However, we were obliged to use both of these datasets in our analysis for different purpose as the census provided age break downs of the populating while WorldPop estimate provided spatial distribution of population.

In assessing the impact of various intervention strategies, it was assumed that effectiveness of a strategy was homogeneous within the area of application, and that anti-viral failure was not observed for a systematic reason, such as hard to reach human or vector populations. This may not necessarily hold true in practice and effort should be made to identify populations which may require a specialized response.

We did not distinguish between serotypes and investigated the combined effect of all circulating strains during the outbreak. The underlying assumption being that no significant difference in transmissibility, symptom severity and reporting probability exists between the circulating subtypes, each having an equal probability to be transmitted and observed. However, genetic analysis of the virus from our patients has shown that two serotypes were largely responsible for the observed outbreak, i.e. serotype 1 and 2. More data on serotype specific immunity would be required to determine if this assumption impacted our model findings.

There are a number of public health messages arising from this study. Overall, our data identifies the dispersed nature of the cases identified, over potentially quite large distances. This suggests that CATI approaches are likely to be more effective if larger areas are targeted for interventions. Our data also points to the essential nature of responding early during an outbreak and the relatively short acting responses expected from mosquito control; with all of these having broad implications for public health responses. In particular, our data suggests that a new antiviral-based prophylactic approach, if it could be developed, would have the potential for reducing the number of cases in outbreaks. This would be beneficial to those who live in regions were dengue virus is endemic and is an important message for public health groups to additionally evaluate when the opportunity arises. This is especially true as regions afflicted by dengue virus are predicted to expand under the effect of global warming and vector displacement.

While further work is needed to better understand how the application of CATI and OTMD strategies can be integrated into routine public health response to dengue outbreaks, our study demonstrates that prophylactic based CATI and OTMD strategies can be effective in reducing dengue morbidity and mortality and would be an effective addition to the tools currently used against dengue infections in outbreak situations.

## Supporting information

S1 Appendix Model description.
(DOCX)

S1 Fig Histograms of model parameters, assuming exponential (A), gravity(B), and radiation(C) movement models over sequential Monte Carlo round (Round 1 = red, Round 2 = green, Round 3 = blue).
(TIF)

S2 Fig Spatial distribution of observed and predicted cases by patch (100mx100m) using (A) Exponential, (B) Gravity, and (C) Radiation movement models.
(TIF)

S3 Fig Frequency of the peak week of the outbreak for each patch (100mx100m) of the four communes, predicted by movement model (A) Exponential, (B) Gravity, (C) Radiation, and (D) as observed in the outbreak data.
(TIF)

S4 Fig Simulation of DENV outbreak, run for 365 days, starting on September 17. Data from two weeks prior to start date were used for model initialisation. Dashed lines show the timepoints that are considered early (day = 1), mid (day = 52), and late (day = 92) in the outbreak. Shaded area indicates confidence intervals.
(TIF)

S5 Fig Predicted daily number of cases from the fitted spatial dengue model using exponential, gravity and radiation-based movement. Black dots indicate daily reported case counts between October 2018 and April 2019. Shaded area indicates confidence intervals.
(TIF)

S1 Table Residual risk factor analysis of previous exposure to DENV.
(DOCX)

S2 Table Final deviation of third round of Monte Carlo rounds and % improvement between first and third Monte Carlo rounds.
(DOCX)

S3 Table Posterior distribution of estimated parameters.
(DOCX)
