# Supplementary material for: Could prophylactic antivirals reduce dengue incidence in a high-prevalence endemic area?
Source: PLoS Negl Trop Dis. 2024 Jul 29;18(7):e0012334. doi: 10.1371/journal.pntd.0012334 (PMC11309446; doi:10.1371/journal.pntd.0012334)
Supplement: S1 Appendix — Model description. (DOCX) [file pntd.0012334.s001.docx]

**S1 Appendix - Model description**

A full description of the model is given in Brady *et al.* [10].

***Mathematical model structure for each patch.***

*Humans (*$h$*) and mosquitoes (*$m$*) are divided into susceptible (*$S$*), exposed (*$E$*), Infectious (*$I$*) and recovered and immune (*$R$*) compartments. Humans can become temporarily immune (*$Rp$*) if treated with prophylactic drugs with effective coverage rate* $r_{d}$ *with protection waning at rate* $c_{d}$*. Infection is controlled by a mosquito-human contact rate (*$\beta$*) after which humans and mosquitoes go through an incubation period (at rates* $\varepsilon_{h}$ *and* $\varepsilon_{m}$ *respectively). Humans then naturally recover after*$1/\gamma$ *days of illness while mosquitoes stay infectious for life. Mosquitoes die at a natural death rate* $\mu_{n}$ *but can also be subject to an additional focal control mortality rate* $\mu_{c}$*. All human population compartments are transient and made up of all individuals who spend time in the patch. Mosquitoes do not move between patches.*

### Transmission dynamics

Mosquitoes within each patch ($i$) exist in one of three states: Susceptible ($S^{m}$), Infected (but not yet infectious) ($E^{m}$) and Infectious ($I^{m}$). Transitions between these states are determined by the following equations:

$${dS^{m}}/{dt} ={\left( \mu_{n}+\mu_{c} \right)\left( S^{m}+E^{m}+I^{m} \right)-\omega S}^{m}-\mu_{n}S^{m}-\mu_{c}S^{m}$$

$${dE^{m}}/{dt}=\omega S^{m}-\mu_{n}E^{m}-\mu_{c}E^{m}-\varepsilon_{m}E^{m}$$

$${dI^{m}}/{dt}=\varepsilon_{m}E^{m}-\mu_{n}I^{m}-\mu_{c}I^{m},$$

Where $\mu_{n}$ and $\mu_{c}$ are the rates mosquito mortality due to natural and extra CATI measures respectively (i.e. we assume vector control measures reduce the number of adult mosquitoes),$\varepsilon_{m}$ is the rate of EIP completion and $\omega$ is the risk of infection for each susceptible mosquito in the patch. Calculating $\omega_{i}$ involves summing the total person hours spent in patch $i$ by both human residents ($T_{i\to i}$) and visitors ($T_{j\to i}$). Infectivity of any given human is a function of the mosquito-human contact rate of patch $i$ ($\beta_{i}$) and the individual infectiousness of each human which is dependent on the number of days since becoming infected ($\theta_{d}$).

$$\omega_{i}= \sum_{j} \left[ T_{j\to i}\left( \frac{\beta_{i}\theta_{d}I_{j,d}^{h}}{N_{j}^{h}} \right) \right]$$

Consistent with observations of viral titre in dengue patients over time, we assume normally distributed infectiousness, peaking at symptom onset (where transmission probability = 1), standard deviation of 2 and constrained to 0 at time of infection and eight days post symptom onset. The timing of peak viremia is determined by a lognormal IIP ($\varepsilon_{h}$).

$$\theta_{d}=N(\varepsilon_{h},2)$$

$$\varepsilon_{h}= Lognormal\left( \mu,\sigma^{2} \right)$$

The additional risk of mortality due to CATI mosquito control efforts ($\mu_{c}$) is applied if the patch ($i$) is within the defined radius ($L$) of an index case ($j$).

$$l_{min}=min(l_{i\to1},\ldots,l_{i\to j})$$

$$f\left( \mu_{c} \right)=\left\{ \begin{aligned} \mu_{c} \mathrm{if} l_{min}\leq L \\ 0 \mathrm{if} l_{min}>L \end{aligned} \right.$$

Infection dynamics in humans were modelled in Susceptible ($S^{h}$), Infectious ($I^{h}$), recovered due to natural infection ($R^{h}$) and temporarily immune due to prophylactic drugs ($R_{p}^{h}$). Transitions between these states are as follows:

$$dS^{h}/dt= rR_{p}^{h}-\varphi_{i}S^{h}-c_{d}S^{h}$$

$$dI^{h}/dt= \varphi_{i}S^{h}-c_{d}I^{h}-I_{d=\frac{1}{\varepsilon_{h}}+\frac{1}{\gamma}}^{h}$$

$${dR^{h}}/{dt}= I_{d=\frac{1}{\varepsilon_{h}}+\frac{1}{\gamma}}^{h}+c_{d}I^{h}$$

$${dR_{p}^{h}}/{dt}= c_{d}S^{h}-rR_{p}^{h}$$

$$S^{h}+I^{h}+R^{h}+R_{p}^{h}=N^{h}$$

Susceptible humans can either be infected in their resident patch or any of the patches they visit:

$$\varphi_{i}=\sum_{j} \left[ T_{i\to j}\frac{\beta_{j}I_{j}^{m}}{\sum_{k} \left( T_{k\to j}N_{k}^{h} \right)} \right]$$

All individuals ($i$) who reside within a distance $L$ of the home location of any detected dengue case ($j$) will receive prophylactic drugs with an effective coverage level $c_{d}$.

$$l_{min}=min(l_{i\to1},\ldots,l_{i\to j})$$

$$f\left( c_{d} \right)=\left\{ \begin{aligned} c_{d} \mathrm{if} l_{min}\leq L \\ 0 \mathrm{if} l_{min}>L \end{aligned} \right.$$

Effective coverage includes barriers to access, adherence, eligibility and efficacy of the prophylactic drug in question. Individuals who are treated with drugs while actively infected are assumed to acquire sterilizing immunity akin to natural infection. Infected individuals remain in the Infectious state for the duration of their IIP + 8 days of disease ($\gamma$) before transitioning to recovered state with sterilizing immunity. Individuals can be detected ($D_{t}^{h}$) at any point in their symptomatic infectious stage (i.e. ${1/\varepsilon}_{h}<d<{1/[\varepsilon}_{h}+\gamma]$) with daily detection probability $\delta$ which is inferred from the case data (N.B. overall probability that a case is detected is therefore $8\delta$):

$$D_{t}^{h}={\delta I}_{t, \varepsilon_{h}<d<[\varepsilon_{h}+8]}^{h}$$

$$\varepsilon_{h,d}= Lognormal\left( \mu,\sigma^{2} \right)$$

The effective reproductive number for each patch was equal to the product of the total number of bites an infectious person receives over their duration of infectiousness ($\theta$), the probability each infected mosquito survives beyond the virus’ EIP [30] and the number of infectious bites delivered to susceptible humans by the infected mosquito population post EIP:

$$R_{t}=\frac{\beta S^{m}\theta}{\bar{N^{h}}} . \frac{\varepsilon_{m}}{\mu_{n}+\varepsilon_{m}} . \frac{\beta\bar{S_{h}}}{\mu_{n}\bar{N_{h}}}$$

$$\bar{N^{h}}=\sum_{j} \left( T_{j\to i}N_{j}^{h} \right)$$

$$\bar{S^{h}}=\sum_{j} \left( T_{j\to i}S_{j}^{h} \right)$$

The model is implemented using a daily time step with events modelled as realisations from stochastic binomial processes. Where multiple additions or subtractions from state compartments existed (e.g. Exposed mosquitoes completing EIP or dying), the order of processes were randomised then sequentially carried out to ensure each mosquito or human had only one outcome and that certain processes were not more likely than others.

We fit the model by minimising mean squared error of the relative distribution of cases across eight equally spaced time periods throughout the epidemic (*T*) and eight quadrants of the study area:

$$d_{x_{\tau}}=T^{rel}+S^{rel}$$

$$T^{rel}=\sum_{T=1}^{T=8} {(D_{T}^{h,observed}-D_{T}^{h,predicted})}^{2}$$

$$S^{rel}=\sum_{Q=1}^{Q=8} \left( D_{Q}^{h,observed}-D_{Q}^{h,predicted} \right)^{2}$$
