# Supplementary figures and images for: Could prophylactic antivirals reduce dengue incidence in a high-prevalence endemic area?
Source: PLoS Negl Trop Dis. 2024 Jul 29;18(7):e0012334. doi: 10.1371/journal.pntd.0012334 (PMC11309446; doi:10.1371/journal.pntd.0012334)

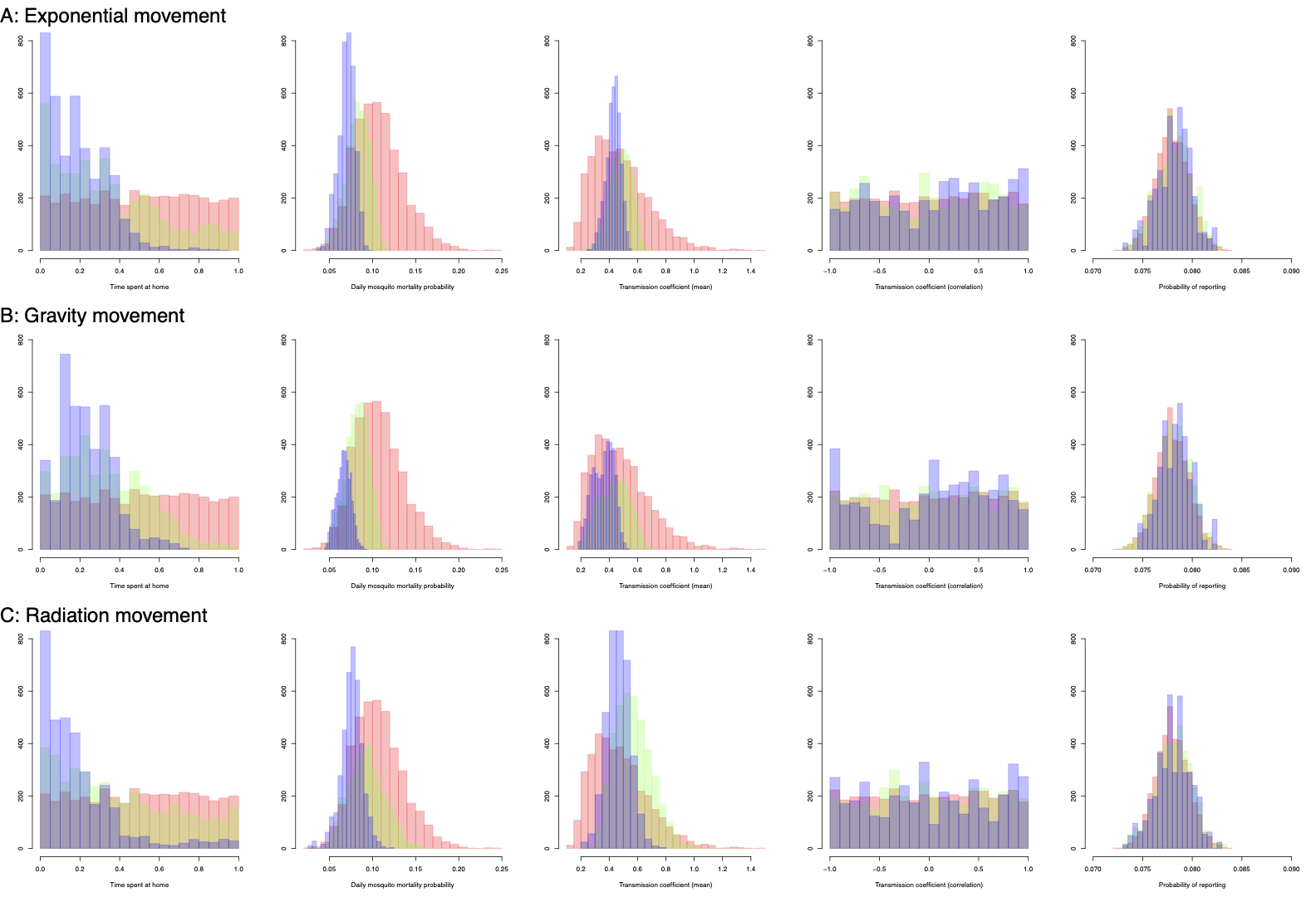

Supplement: S1 Fig — Histograms of model parameters, assuming exponential (A), gravity(B), and radiation(C) movement models over sequential Monte Carlo round (Round 1 = red, Round 2 = green, Round 3 = blue). (TIF) [file pntd.0012334.s002.tif]

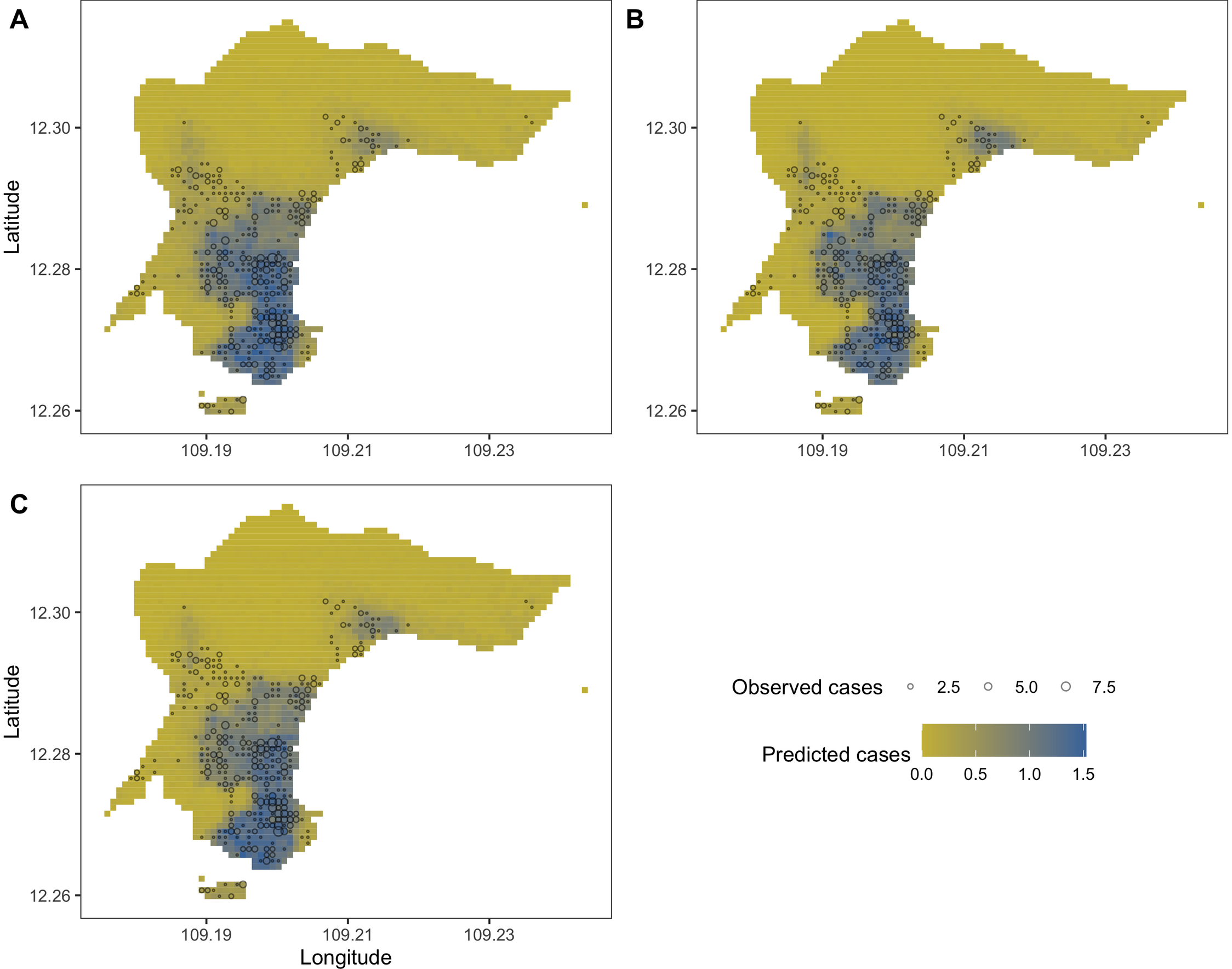

Supplement: S2 Fig — Spatial distribution of observed and predicted cases by patch (100mx100m) using (A) Exponential, (B) Gravity, and (C) Radiation movement models. (TIF) [file pntd.0012334.s003.tif]

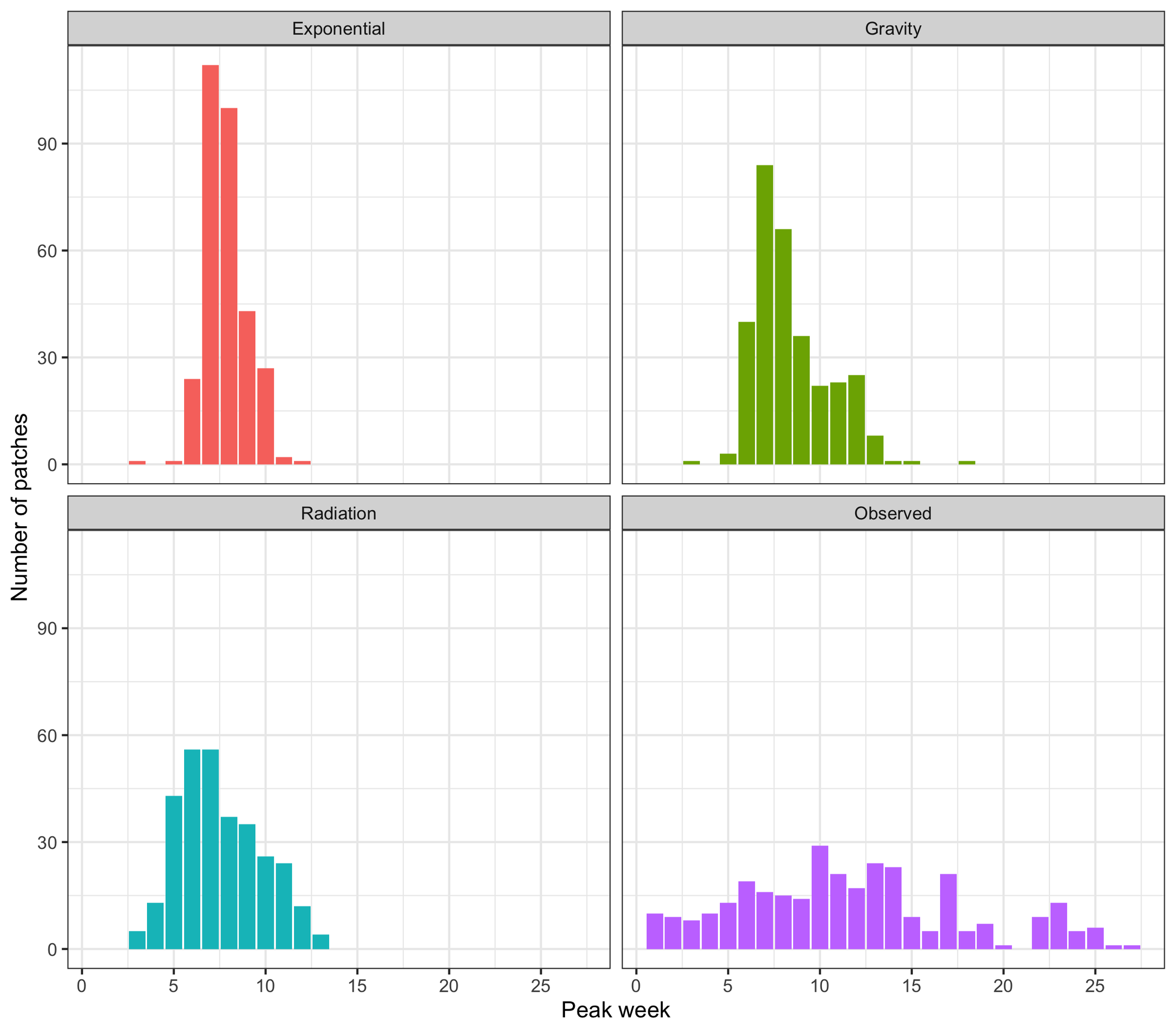

Supplement: S3 Fig — Frequency of the peak week of the outbreak for each patch (100mx100m) of the four communes, predicted by movement model (A) Exponential, (B) Gravity, (C) Radiation, and (D) as observed in the outbreak data. (TIF) [file pntd.0012334.s004.tif]

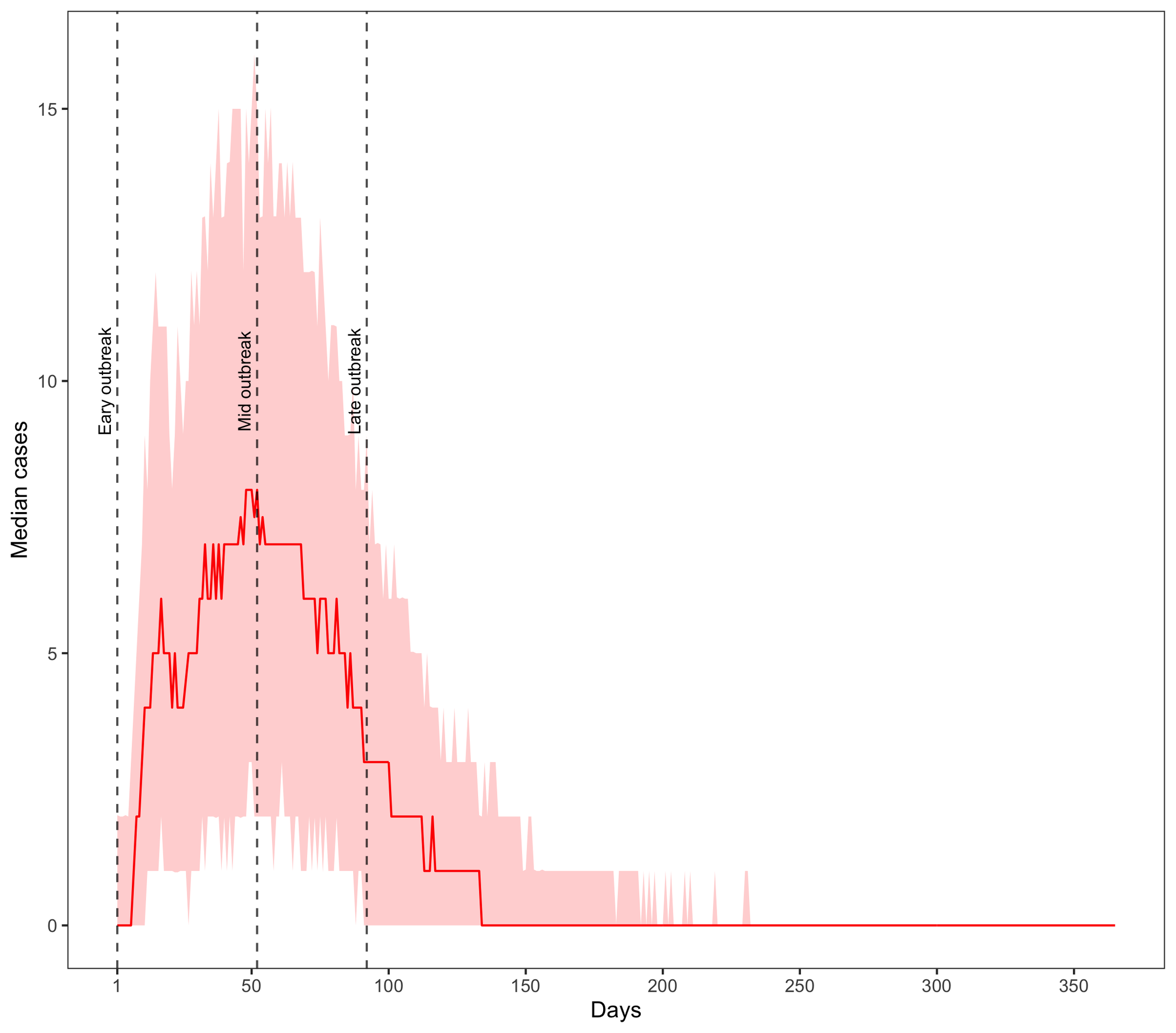

Supplement: S4 Fig — Simulation of DENV outbreak, run for 365 days, starting on September 17. Data from two weeks prior to start date were used for model initialisation. Dashed lines show the timepoints that are considered early (day = 1), mid (day = 52), and late (day = 92) in the outbreak. Shaded area indicates confidence intervals. (TIF) [file pntd.0012334.s005.tif]

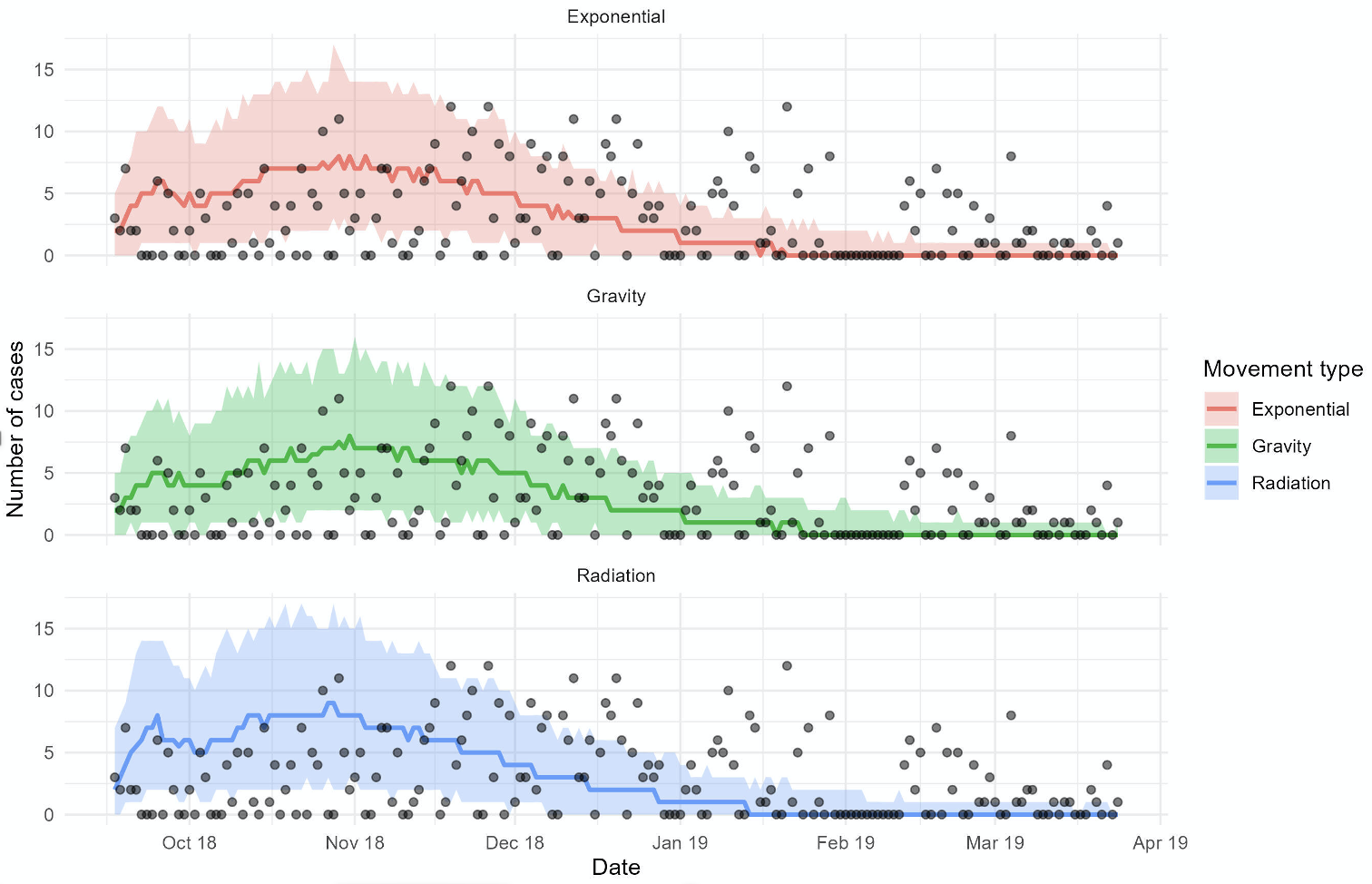

Supplement: S5 Fig — Predicted daily number of cases from the fitted spatial dengue model using exponential, gravity and radiation-based movement. Black dots indicate daily reported case counts between October 2018 and April 2019. Shaded area indicates confidence intervals. (TIF) [file pntd.0012334.s006.tif]
