# Supplementary material for: Could prophylactic antivirals reduce dengue incidence in a high-prevalence endemic area?
Source: PLoS Negl Trop Dis. 2024 Jul 29;18(7):e0012334. doi: 10.1371/journal.pntd.0012334 (PMC11309446; doi:10.1371/journal.pntd.0012334)
Supplement: S1 Table — Residual risk factor analysis of previous exposure to DENV. (DOCX) [file pntd.0012334.s007.docx]

**S1 Table - Residual risk factor analysis of previous exposure to DENV**

| Risk Factor | Sample size  n(N) | Residual Risk | 95% CI | |
| --- | --- | --- | --- | --- |
|  |  |  | Low | High |
| Sex |  |  |  |  |
| Male | 218 (508) | Reference | | |
| Female | 290 (508) | 2.8E-02 | -1.0E-02 | 6.6E-02 |
| HH* Occupation |  |  |  |  |
| Office Professional | 58 (508) | Reference | | |
| Worker | 124 (508) | 1.1E-02 | -1.5E-02 | 3.8E-02 |
| Other | 43 (508) | 1.0E-02 | -1.4E-02 | 3.5E-02 |
| Chronic | 18 (508) | -9.9E-02 | -2.3E-01 | 3.2E-02 |
| HH* Log income | 508 (508) | -3.0E-02 | -6.7E-02 | 6.1E-03 |
| HH* Education |  |  |  |  |
| No school | 22 (508) | Reference | | |
| High school | 446 (508) | 8.1E-03 | -6.2E-02 | 7.8E-02 |
| Post-secondary | 40 (508) | -2.3E-02 | -1.2E-01 | 7.2E-02 |
| Anyone smokes in house | 264 (508) | 8.3E-03 | -2.8E-02 | 4.4E-02 |
| House size (log(m)) | 507 (507) | -6.3E-03 | -3.7E-02 | 2.4E-02 |
| Ever hospitalized | 82 (508) | -2.2E-03 | -5.5E-02 | 5.1E-02 |
| HH* Head |  |  |  |  |
| Father | 206 (508) | Reference | | |
| Mother | 88 (508) | 2.9E-02 | -2.1E-02 | 7.9E-02 |
| Grandparent | 108 (508) | -4.4E-02 | -1.1E-01 | 2.7E-02 |
| Other | 42 (508) | -2.9E-02 | -9.9E-02 | 4.2E-02 |
| Self | 64 (508) | 2.0E-04 | -3.7E-02 | 3.8E-02 |
| Japanese encephalitis vaccine | 6 (244) | -6.3E-02 | -1.6E-01 | 3.2E-02 |
