# Supplementary material for: Could prophylactic antivirals reduce dengue incidence in a high-prevalence endemic area?
Source: PLoS Negl Trop Dis. 2024 Jul 29;18(7):e0012334. doi: 10.1371/journal.pntd.0012334 (PMC11309446; doi:10.1371/journal.pntd.0012334)
Supplement: S2 Table — Final deviation of third round of Monte Carlo rounds and % improvement between first and third Monte Carlo rounds. (DOCX) [file pntd.0012334.s008.docx]

**S2 Table – Final deviation of third round of Monte Carlo rounds and % improvement between first and third Monte Carlo rounds**

| **Movement Model** | **Final Deviation** | **% improvement** | | |
| --- | --- | --- | --- | --- |
|  |  | **Time** | **Space** | **Overall** |
| **Exponential** | 0.42(0.34, 0.52) | 39.3 | 49.2 | 41.9 |
| **Gravity** | 0.42 (0.34, .51) | 40.2 | 45.8 | 41.7 |
| **Radiation** | 0.48 (0.41, 0.57) | 34.3 | 32.5 | 33.8 |
