# Supplementary material for: Could prophylactic antivirals reduce dengue incidence in a high-prevalence endemic area?
Source: PLoS Negl Trop Dis. 2024 Jul 29;18(7):e0012334. doi: 10.1371/journal.pntd.0012334 (PMC11309446; doi:10.1371/journal.pntd.0012334)
Supplement: S3 Table — Posterior distribution of estimated parameters. (DOCX) [file pntd.0012334.s009.docx]

**S3 Table – Posterior distribution of estimated parameters**

| **Parameter** | **Exponential**  mean(95% CrI) | **Gravity**  mean(95% CrI) | **Radiation**  mean(95% CrI) |
| --- | --- | --- | --- |
| **Distance decay parameter controlling human movement** | 0.19 (0.10, 0.48) | 0.23 (0.029, 0.56) | 0.17 (95% CrI: 0.0077,0.82) |
| **Mean vector-to-human and human-to-vector transmission rate** | 0.43 (0.32, 0.52) | 0.36 (95% CrI: 0.22, 0.48), | 0.46 (0.31, 0.62) |
| **Spatial correlation of vector-to-human transmission rate** | 0.080 (-0.94, 0.99) | 0.48 (-1.0 0.93) | 0.021 (95% CrI: -0.98, 0.99) |
| **Daily mortality rate of a mosquito** | 0.070 (0.050, 0.086) | 0.067(0.050, 0.082) | 0.076 (0.049, 0.099) |
| **Daily probability of a DENV infected individual being detected and reported as a case** | 0.0098 (0.0093, 0.010) | 0.0098 (0.0094, 0.010) | 0.0098 (0.0093, 0.010) |
